# The blood level of thioredoxin 1 as a supporting biomarker in the detection of breast cancer

**DOI:** 10.1186/s12885-021-09055-1

**Published:** 2022-01-03

**Authors:** Youn Ju Lee, Young Kim, Bo Bae Choi, Je Ryong Kim, Hye Mi Ko, Kyoung Hoon Suh, Jin Sun Lee

**Affiliations:** 1 Department of Surgery, Chungnam National University Sejong Hospital, 20, Bodeum 7-ro, Sejong, South Korea; 2 E&S Healthcare, 11-3, Techno 1-ro, Yuseong-gu, Daejeon, South Korea; 3 Department of Surgery, College of Medicine, Yonsei University, 262 Seongsan-no, Seodaemun-gu, Seoul, South Korea; 4 Department of Radiology, Chungnam National University Hospital, 282, Munhwa-ro, Jung-gu, Daejeon, South Korea; 5 Department of Surgery, Chungnam National University Hospital, 282, Munhwa-ro, Jung-gu, Daejeon, South Korea; 6 Department of Surgery and Research Institute for Medicinal Sciences, Chungnam National University, School of Medicine, 266, Munhwa-ro, Jung-gu, Daejeon, South Korea; 7 Department of Life Science and Technology, Pai Chai University, 11-3, Techno 1-ro, Yuseong-gu, Daejeon, South Korea

**Keywords:** Thioredoxin 1, Breast Cancer, Diagnostics, Mammography, Sensitivity, Specificity, Early diagnosis, Diagnostic interval, Blood, Biomarker

## Abstract

**Background:**

There is a long-time unmet need for a means to detect breast cancer (BC) using blood. Although mammography is accepted as the gold standard for screening, a blood-based diagnostic can complement mammography and assist in the accurate detection of BC in the diagnostic process period of early diagnosis. We have previously reported the possible use of thioredoxin 1 (Trx1) in serum as a novel means to detect BC. In the present study, we validated the clinical utility of Trx1 to identify BC by testing sera from biopsy-confirmed cancer patients and women without cancer.

**Methods:**

We have generated monoclonal antibodies against Trx1 and developed an ELISA kit that can quantitate Trx1 in sera. The level of Trx1 was determined in each serum from women without cancer (*n* = 114), as well as in serum from patients with BC (*n* = 106) and other types of cancers (*n* = 74), including cervical, lung, stomach, colorectal, and thyroid cancer. The sera from BC patients were collected and classified by the subjects’ age and cancer stage. In addition to the Trx1 levels of BC patients, several pathological and molecular aspects of BC were analyzed. Test results were retrospectively compared to those from mammography. Each test was duplicated, and test results were analyzed by ROC analysis, one-way ANOVA tests, and unpaired t-tests.

**Results:**

The mean level of Trx1 from women without cancer was 5.45 ± 4.16 (±SD) ng/ml, that of the other malignant cancer patient group was 2.70 ± 2.01 ng/ml, and that from the BC group was 21.96 ± 6.79 ng/ml. The difference among these values was large enough to distinguish BC sera from non-BC control sera with a sensitivity of 97.17% and specificity of 94.15% (AUC 0.990, *p* < 0.0001). Most Trx1 levels from BC patients’ sera were higher than the cut-off value of 11.4 ng/ml regardless of age, stage, histological grade, type, and specific receptors’ expression profile of BC. The level of Trx1 could rescue women from most cases of misread or incomplete mammography diagnoses.

**Conclusion:**

These results indicated that the blood level of Trx1 could be an effective and accurate means to assist the detection of BC during the early diagnosis period.

## Background

It is well-known that BC comprises the highest incidence of tumors in women [1, 2]. A worldwide study showed that, in 2020, an estimated 2.2 million women were newly diagnosed with BC, and more than 684,996 women died of BC [2]. In developing countries, despite a lower reported incidence of BC, the mortality rate is generally higher. This is likely due to delayed presentation, late stage at diagnosis, and limited access to treatment. This reflects both the risk factors from their lifestyles and the availability and utility of proper BC screening [3–6].

Breast cancer is generally diagnosed by screening systems including mammography and ultrasound scanning. The World Health Organization (WHO) recommended mammography as the primary screening tool for BC because it has demonstrated its utility to reduce BC mortality compared to other imaging diagnostic methods [7, 8]. Although it is known that mammography significantly reduces mortality in women over 50 years of age, it is not as helpful for younger women [9, 10]. The current most advanced digital mammography systems are not perfect, with values of sensitivity and specificity to detect BC of 90% or below [11, 12]. When it comes to dense breasts, the performance of mammography shows even lower numbers [12, 13]. Although the mammography system is currently regarded as the best method to detect BC as early as possible, it also has disadvantages. Most mammography equipment is installed in specific hospitals as immobile instruments, so women must take a trip to visit a facility where the machine is available. Physical and mental discomfort experienced in prior mammography sessions also causes hesitation about or even the foregoing of mammography. The limited accessibility to mammography in developing countries is another major obstacle to the early detection of BC. Therefore, it is desirable to have a means that complements mammography and thus mitigates current problems and limitations of the mammography-oriented diagnostic system.

In addition to the screening of BC by mammography, the discovery of biomarkers by analyzing various body fluids has drawn attention for its potential usage in easy and rapid detection of cancer. A study of 1005 patients to evaluate the clinical utility of analyzing specific circulating proteins and cell-free DNA in blood for the early detection of eight types of cancer has been undertaken [14]. Although it showed sensitivities for certain types of cancer ranging from 69 to 98%, that for BC was little more than 30%. Recently, another massive study with 6689 participants attempted to determine whether targeted methylation analysis of cell-free DNA was effective for the early detection of certain types of cancer [15]. The results indicated a sensitivity for twelve cancer types of 67.3%, but BC was not included. Despite tremendous investment in liquid biopsy technology as an innovative means to discover novel biomarkers that are promising for the detection of BC, there is no obvious candidate. According to an early cancer diagnosis guideline from WHO, the role of screening of BC for detection of unrecognized cancer or pre-cancerous lesions in an apparently healthy target population is relatively well served by mammography [16]. On the other hand, the early BC diagnosis step which follows the screening step still needs more means for better and timely diagnosis. The early diagnosis is defined as the early identification of cancer in patients who have symptoms and signs consistent with cancer. Therefore, it will be worthy of adding a new approach to help early diagnosis of cancer.

We have reported that there is a close relationship between BC occurrence and the serum level of a member of the antioxidative protein families: thioredoxin 1 (Trx1) [17, 18]. The blood levels of Trx1 from BC patients were higher than those from women without cancer. Its sensitivity and specificity to detect BC were higher than those of commonly using biomarkers, CA15-3 and CEA. Since the blood level of Trx1 could be a novel standard to evaluate the current risk of BC, we have investigated its clinical utility as a biomarker to help detect BC during the early diagnostic step for women who have shown symptoms or cancerous masses in their breasts. This step is different from screening for and identification of cancer at the earliest possible opportunity and link to diagnosis and treatment without delay [16]. Therefore, we tested sera from women who had been confirmed to have BC by biopsy, from women without cancer, and from women with other types of cancer as well. The level of Trx1 in blood was analyzed according to the age of BC patients, as well as the stage, types, and grade of their BC. A comparison analysis with other types of cancer to examine the selectivity of Trx1 for BC was also carried out. In addition, the Trx1 level was analyzed, along with mammography data, to assess how well the Trx1 level could complement or assist mammography for the better detection of BC. It was hypothesized that the level of Trx1 in blood was likely to be a novel and effective means for the early diagnosis of BC.

## Methods

### Study design

This cross-sectional and retrospective study assessed the clinical utility of the Trx1 level in blood to assist in the detection of BC in the early diagnosis period [16]. Since early diagnosis is the step to diagnose BC in women who have cancer-related symptoms or masses in their breasts detected from a prior screening step, we tested sera from women who had been confirmed to have BC. Blood was collected before surgery or any type of treatment for the confirmed BC patients.

Among the BC patient candidates for the study who were confirmed to have BC by biopsy during the present study, only those whose prior mammograms were archived at Chungnam National University Hospital (CNUH) were selected. The levels of Trx1 of the blood from these selected BC patients (*n* = 106) were analyzed. The BC patients were grouped according to their clinical information and BC characteristics. Attention was paid to distribute a reasonable number of patients to each group. Women with other types of cancer (cervical, *n* = 17; lung, *n* = 30; stomach, *n* = 9; colorectal, *n* = 14; thyroid, *n* = 4) were selected if their blood samples were available from CNUH after a physician’s final confirmation of the corresponding cancer (Table 1). Since the number of patients with other types of cancer whose deposited sera and necessary clinical information were available from the Human Body Resources Bank of CNUH was limited, a total of 74 patients were all that could be included in the present study. Therefore, acknowledging that the number was low, these subjects were combined and analyzed as one group. Although the number of blood samples from patients with each cancer type seems small, the main reason to include other cancer types was to see the potential of the Trx1 level to differentiate BC from other types of cancer. The primary purpose of the present study was to assess the clinical utility of Trx1 in breast cancer diagnostic procedure. Another well-organized study with a larger number of samples from various types of cancer is necessary in the future to investigate the Trx1 levels of each other type of cancer. Also recruited were women without cancer (*n* = 114) who exhibited no cancer history or breast-related disease, confirmed by a physician during normal periodic physical examinations. To assess the Trx1 level’s accuracy and capacity to complement the imaging diagnosis of BC, Trx1 test results were compared with the first readings of diagnostic mammograms, which were collected afterward. The level of Trx1 from each serum was determined by an ELISA kit, DxMe BC (E&S Healthcare, BCE01, Korea).

**Table 1:** Participants in the study

Participants^**a**^		
Serum sources	Trx1 (95% CI)	No. (***n*** = 106)
Women without cancer	5.45 (4.67 to 6.22)	114
Breast cancer	21.96 (20.65 to 23.27)	106
Lung cancer	2.34 (1.66 to 3.02)	30
Cervical cancer	2.51 (1.67 to 3.36)	17
Colorectal cancer	3.13 (1.87 to 4.38)	14
Stomach cancer	3.64 (1.34 to 5.94)	9
Thyroid cancer	2.65 (0.05 to 5.25)	4

^a^ Sera from designated participants were obtained from Chungnam National University Hospital with ethical committee approval and patients’ informed consent

Participant recruitment and blood sample collection protocols were approved (CNUH2017-12-035) by an ethical committee from Chungnam National University Hospital in Daejeon, Korea, with patients’ informed consent, and study with the blood samples was conducted in accordance with the relevant guidelines and regulations.

### Serum preparation

The blood samples used in this study were collected and deposited at the Human Body Resources Bank of CNUH. A designated phlebotomist withdrew blood from the cephalic vein into serum separating tubes (BD Vacutainer, SST™ II Advance Plus Blood Collection Tubes). After standing at room temperature for blood clotting and maturation for 6 hours, the serum separating tubes were centrifuged at 1000 x g for 15 min. The serum portion of the supernatant was collected and transferred to cryogenic tubes. The sera were stored at − 70 °C until use. Because of the biochemical characteristics of the detection antibody used in the Trx1-quantitating ELISA kit, which exhibited a higher affinity to the oxidized dimeric form of Trx1 than to the reduced monomeric one, the test was optimized to mature blood for 6 hours to achieve the highest levels of dimeric Trx1. This process helped get the highest detection of total Trx1. According to validation tests of this kind of maturation process, Trx1 levels in blood from both BC patients and women without cancer increased during a maturation period of 6 hours, then stabilized. The largest difference between the Trx1 levels detected by the antibody in blood from BC patients and women without cancer was achieved at that time point. The properly stored serum samples from this maturation process did not show any change in the Trx1 level detected by the ELISA kit for more than a year.

### Quantitation of Trx1 in serum

We have generated a pair of monoclonal antibodies against Trx1. Using these antibodies, the DxMe BC ELISA kit (E&S Healthcare) was developed to quantitate Trx1 from sera. The kit was based on sandwich ELISA technology, and the test procedure in the present study followed the instructions of the kit, which were modified from protocols described elsewhere [17, 19]. Single-blind tests were performed for each serum thus the disease status of the serum samples was not known to the analysts. The quality of the ELISA kit was assessed by CLSI (Clinical and Laboratory Standard Institute) guidelines EP05-A3 and EP15-A3. Its coefficient of variation (CV) of reproducibility and repeatability (e.g., between-lots, between-testers, and between locations) was within 10%. Test sera from control groups and BC patients were tested by kits from the same lot. The level of Trx1 in serum was calculated from the standard curve that was generated using pure recombinant human Trx1 protein.

### Comparison analysis with mammography

In order to evaluate the ability of the Trx1 test to complement or mitigate the limitations of current mammography, a comparison study was carried out. The blood level of Trx1 of each group of women without breast cancer and breast cancer patient was measured as described above and analyzed along with a radiologic report of the corresponding mammogram by a breast radiologist with 12 years of experience. Among breast cancer patients who had been confirmed to have BC by biopsy, those who had taken their mammogram at CNUH were finally selected for the study. For women without cancer who had been confirmed not to have any cancer history or symptoms of the breasts, those who had undergone mammography at CNUH were recruited. Samples of patients who had been confirmed to have other types of cancer by biopsy were collected. Many of the women without cancer underwent mammography at other specialized clinics, so their mammograms were not accessible in this study. Therefore, in the end, mammograms from 42 women without cancer and 103 BC patients were analyzed together with theirs blood levels of Trx1.

### Statistical analysis

The sensitivity and specificity were calculated by ROC curve analysis with a predetermined cut-off value. The data were further analyzed by Kruskal-Wallis tests, one-way ANOVA tests, and unpaired t-tests when necessary. Statistical analysis was performed using the MedCalc (ver.19.1.5, MedCalc Software Ltd.) and Prism 6 (GraphPad) software. It was regarded as statistically significant when *p* < 0.001.

## Results

### Breast cancer patients

A total of 106 patients who had been confirmed to have BC by biopsy were recruited, and their mean age was close to 50. Most BC patients were at stage 2 (*N* = 50, 47.2%) and the rest of them were at stage 1 (*n* = 37, 34.9%), stage 3 (*n* = 15, 14.2%) and stage 0 and 4 (*n* = 2 each, 1.9% each) (Table 2). More than 86% of the patients had invasive ductal carcinoma (IDC, *n* = 92) while patients with each of the other types of BC, such as ductal carcinoma in-situ (DCIS), invasive lobular carcinoma (ILC), invasive micropapillary carcinoma (IMPC), mucinous carcinoma (MC), and invasive tubular carcinoma (ITC), were low in number, from 1 to 5.

**Table 2:** Correlation between Trx1 level and clinicopathological characteristics in breast cancer patients and women without cancer

**Correlation between Trx1 level and age in breast cancer patients and women without cancer**					
**Variable**	**Age**	**Trx1 (95% CI)**	**No.**	**%**	***P*** **Value**
Women without cancer	30s	3.16(1.16 to 5.16)	3	2.6	0.6689
(*n* = 114)	40s	5.99(4.06 to 7.91)	14	12.3	
	50s	6.31(4.67 to 7.95)	30	26.3	
	60s	4.84(3.55 to 6.12)	36	31.6	
	70s	5.29(3.44 to 7.15(	29	25.4	
	80s	5.25(NA)	2	1.8	
Breast cancer patients	30s	22.60 (16.0 to 29.2)	7	6.6	0.705
(*n* = 106)	40s	22.85 (20.5 to 25.2)	47	44.3	
	50s	20.75 (19.22 to 22.27)	42	39.6	
	60s	22.44 (17.0 to 27.82)	10	9.4	
**Correlation between Trx1 level and clinicopathological characteristics in breast cancer patients**^**a**^					
**Variable**		**Trx1(95% CI)**	**No. (*n*** **= 106)**	**%**	***P*** **Value**
Histologic grade	1	22.08 (19.61 to 24.55)	24	22.6	0.8422
	2	21.19 (19.54 to 22.84)	51	48.1	
	3	23.14 (19.95 to 26.32)	31	29.3	
Histologic subtypes	DCIS	22.25 (NA)	2	1.9	0.5083
	IDC	22.00 (20.54 to 23.45)	92	86.8	
	ILC	24.63 (19.78 to 29.48)	5	4.7	
	MC	20.41 (13.78 to 27.03)	5	4.7	
	IMPC	19.07 (NA)	1	0.9	
	ITC	15.55 (NA)	1	0.9	
Molecular subtypes	Luminal A	20.18 (18.44 to 21.92)	47	44.3	0.1043
	Luminal B	23.31 (20.69 to 25.92)	37	34.9	
	TNBC	24.45 (20.92 to 27.98)	13	12.3	
	HER2-enriched	22.15 (17.43 to 26.87)	9	8.5	
TNM Stage	0	22.25 (NA)	2	1.9	0.4383
	1	21.64 (19.48 to 23.8)	37	35	
	2	21.94 (20.22 to 23.66)	50	47.2	
	3	21.86 (16.46 to 27.26)	15	14.2	
	4	28.97 (NA)	2	1.9	
Ki67	< 15%	21.52 (20.06 to 22.97)	49	46.2	0.7368
	≥15%	22.34 (20.22 to 24.47)	57	53.8	

^a^ The clinical information of breast cancer patients was classified according to breast cancer stage, histological grade, proliferation activity, type, and molecular subtype. *DCIS* ductal carcinoma in situ, *IDC* invasive lobular carcinoma, *MC* mucinous carcinoma, *IMPC* invasive micropapillary carcinoma, *ITC* invasive tubular carcinoma, *TNBC* triple-negative breast cancer

Almost half of the patients exhibited histological grade 2 (*N* = 51, 48.1%), and combined grades 1 and 3 showed similar numbers. Luminal A and B composed almost 80% of patients, and triple negative breast cancer (TNBC, 12.3%) and HER2-enriched (8.5%) comprised the rest. More than 53% of the patients presented with high cancer cell proliferation activity (≥15%) estimated by the Ki67 test that was performed along with the biopsy. Each BC patient went through necessary examinations, such as mammography, ultrasonic scanning, immunohistochemistry, gene expression of specific receptors, or MRI when necessary.

### Ability of blood Trx1 level to identify breast cancer

**Fig. 1:**
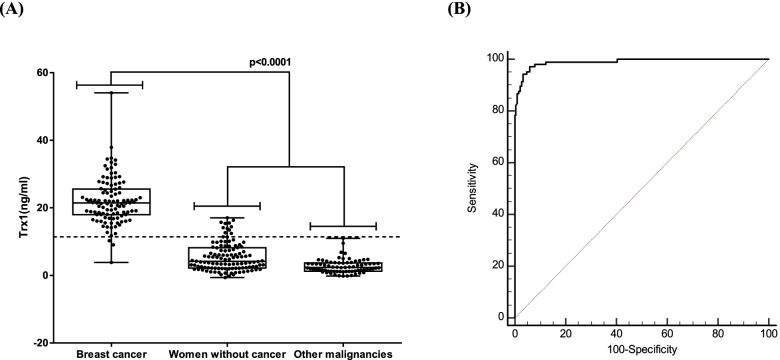
Higher blood level of Trx1 in BC patients compared with women without cancer and other types of cancer patients. **A** Separation of BC patients from non-BC control, including women without cancer and patients from 5 different types of cancer by blood level of Trx1. The dotted line indicates the cut-off value of Trx1 level (11.4 ng/ml). **B** ROC curve analysis to determine the ability of blood Trx1 level to differentiate BC from non-BC group. The sensitivity and specificity were 97.17 and 94.15%, respectively, with an AUC of 0.990 ± 0.005

The Trx1 level of sera estimated by the DxMe BC ELISA kit showed good differentiation between BC patients and the non-BC group that included women without cancer and patients with other types of cancer (Fig. 1). Twelve out of the 188 non-BC subjects (6.38%) had a level over the cut-off value (11.4 ng/ml), and three out of the 106 BC patients (2.91%) were below the cut-off. The mean value of the Trx1 levels from women without cancer was 5.45 ± 4.16 ng/ml, while that of patients with other types of cancer was 2.70 ± 2.01 ng/ml, and that from BC patients was 21.96 ± 6.79 ng/ml. The difference of mean values of serum Trx1 levels among women without cancer, patients with other types of cancer, and BC patients was large enough to distinguish BC from non-BC cases. The sensitivity and specificity of the test, determined by ROC curve analysis, were 97.17 and 94.15%, respectively. The area under curve (AUC) was 0.990.

Even though the number of each type of other cancer was relatively low, reducing the ability to assign statistical meaning to the data, the results revealed a difference in Trx1 levels between the two groups large enough to differentiate BC from other types of cancer, indicating that the blood level of Trx1 was likely to identify BC specifically.

### Effect of age

**Fig. 2:**
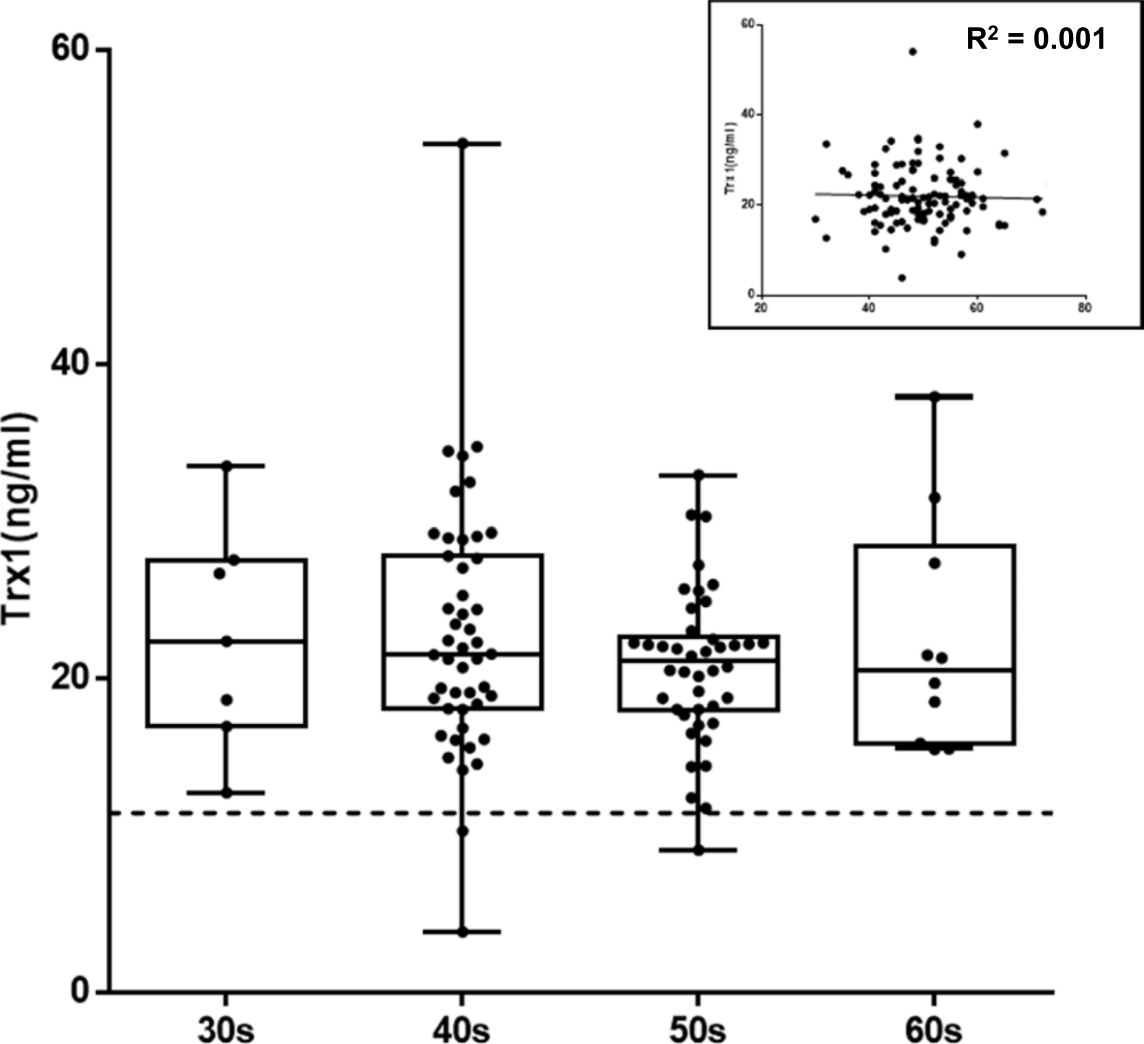
The effect of age on the level of blood Trx1. Breast cancer patients were divided into age groups of the 30s, 40s, 50s, and 60 and over, and their blood levels of Trx1 were compared. The enclosed box is a linear regression of Trx1 level dependency on age showing R^2^ of 0.001. The dotted line indicates the cut-off value of Trx1 level (11.4 ng/ml)

Breast cancer patients were grouped according to their age group, such as 30’s, 40’s, 50’s, and 60 and over, and each serum was tested to quantitate Trx1 and assess the effect of age (Fig. 2). The mean value of Trx1 in sera from BC patients in their 30’s was 22.60 ± 7.14 ng/ml, and those of patients in their 40’s, 50’s, and 60’s and over was 22.85 ± 7.99, 20.75 ± 4.90, and 22.44 ± 7.53 ng/ml, respectively (Table 2). Interestingly, the mean values did not differ much with age, whereas the BC incidence rate in general went up as women got older. The average level of Trx1 from BC patients of all ages was 22.16 ± 6.89 ng/ml. The mean age of all patients was close to 50, which is in the age range showing the highest incidence of BC in Korea [20]. Breast cancer patients under and over the age of 50 showed similar levels of blood Trx1. The R-squared (R^2^ = 0.001) from linear regression of the age-base analysis showed a very low value indicating that it is likely that there is no relationship between the level of Trx1 and patient age.

### Effect of breast cancer types and molecular subtypes

**Fig. 3:**
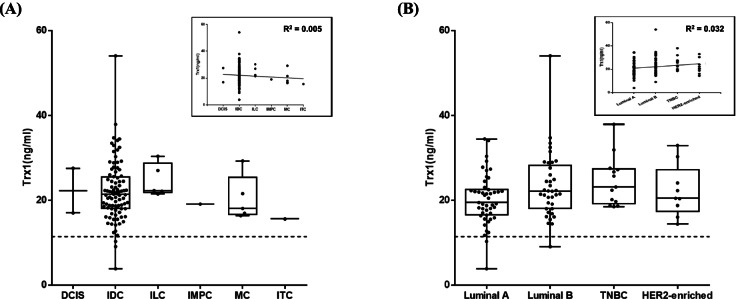
The effect of breast cancer types on the blood level of Trx1. **A** The effect of pathological types of breast cancer. DCIS, ductal carcinoma in-situ; IDC, invasive ductal carcinoma; ILC, invasive lobular carcinoma; IMPC, invasive micropapillary carcinoma; MC, mucinous carcinoma; ITC, invasive tubular carcinoma. **B** The effect of molecular subtypes of breast cancer on the level of blood Trx1. TNBC, triple negative breast cancer. The enclosed boxes are linear regression of Trx1 level dependency on BC type and subtype. The dotted line indicates the cut-off value of Trx1 level (11.4 ng/ml)

The levels of Trx1 were analyzed according to the types of BC, such as DCIS, IDC, ILC, IMPC, MC, and ITC to examine whether there was any correlation between BC type and Trx1 level (Fig. 3A). Most patients had IDC (*n* = 92), which had a mean level of Trx1 of 22.00 ± 7.04 ng/ml. The levels were 24.63 ± 3.91 ng/ml for ILC (*n* = 5), 20.41 ± 5.34 ng/ml for MC (*n* = 5), 22.25 ± 7.47 ng/ml for DCIS (*n* = 2), 19.07 ng/ml for IMPC (*n* = 1), and 15.55 ng/ml for ITC (*n* = 1). All BC types showed levels ranging from 20.41 ± 5.34 ng/ml to 24.63 ± 3.91 ng/ml. In spite of an insufficient number of sera from IMPC and ITC, as well as the relatively wide spread of the Trx1 levels in IDC, the mean values of all types of BC were higher than the cut-off value and exhibited a very low r^2^ value, indicating that there was no effect of BC type on the level of Trx1for BC detection.

In addition, the effect of molecular subtypes of BC was also investigated (Fig. 3B). Luminal A and B were known to be the most common subtypes of BC, which showed blood Trx1 levels of 20.18 ± 5.92 ng/ml and 23.31 ± 7.85 ng/ml, respectively. Other subtypes, including TNBC and HER2-enriched, also presented similar values to luminal A and B. The average level of Trx1 was 22.52 ± 6.44 ng/ml, which was much higher than the cut-off value. It indicated that there was no difference in Trx1 level in different molecular subtypes of BC.

### Effect of specific receptor expression profile

**Fig. 4:**
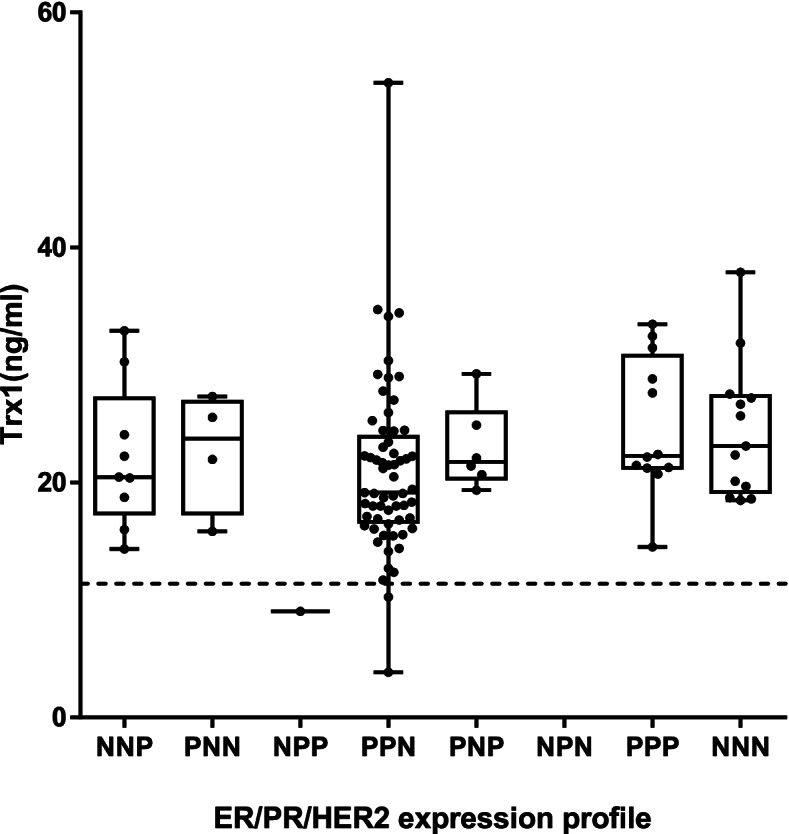
The effect of hormone receptor expression profile on the blood level of Trx1. Trx1 levels from breast cancer patients were analyzed by the expression profiles of estrogen receptor (ER), progesterone receptor (PR), and human epidermal growth factor receptor 2 (HER2). Three-letter remarks on x-axis indicate positive (P) or negative (N) expression of ER, PR, and HER2 in order. For example, PPN indicates ER^+^, PR^+^ HER2^−^. The dotted line indicates the cut-off value of Trx1 level (11.4 ng/ml)

As specific receptor expressions are associated with specific pathological and genetic implications and thus have differing indications for the treatment of BC, it is important to see whether the blood level of Trx1 is influenced by the receptor expression patterns. Therefore, the blood level of Trx1 was estimated from sera exhibiting the different expression profiles of ER, PR and HER2 (Fig. 4). Each receptor expression was assessed during biopsy. Almost 58% of BC patients had hormone receptor expression profiles of ER^+^, PR^+^, and HER2^−^ (PPN). This group showed an average Trx1 level of 20.91 ± 7.30 ng/ml. The triple negative case (ER^−^, PR^−^, HER2^−^, NNN) came second in number (11.3%) and indicated a level of 24.45 ± 5.84 ng/ml. The triple positive (ER^+^, PR^+^, HER2^+^, PPP) consisted of 11.3% of the patients and showed an average level of Trx1 of 24.80 ± 5.83 ng/ml. Only one patient exhibited the ER^−^. PR^+^, HER2^+^ (NPP) expression, so no scientific interpretation could be made. Since the patients with certain specific receptor expression profiles were hard to recruit due to low incidence, the corresponding numbers of such patients in the study were low. However, their levels of Trx1 ranged from 22.15 ± 6.14.13 ng/ml to 22.94 ± 3.59 ng/ml when the one patient with NPP was excluded, which were much higher than the cut-off value. These results indicated that the expression pattern of ER, PR and HER2 did not influence the level of Trx1 in sera of BC patients.

### Effect of breast cancer stage and grade

**Fig. 5:**
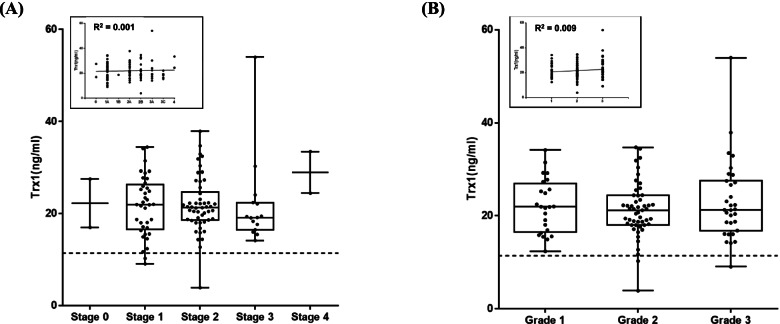
The effect of breast cancer stage and grade on the blood level of Trx1. Trx1 levels from breast cancer patients were analyzed by stage and grade of the cancer. **A** Effect of breast cancer stage. The number of patients at each stage were 2 at stage 0, 37 at stage 1, 50 at stage 2, 15 at stage 3, and 2 at stage 4. **B** Effect of breast cancer grade that was assessed by biopsy. The number of patients at each grade were 24 at grade 1, 51 at grade 2, and 31 at grade 3. The enclosed boxes are linear regression of Trx1 level dependency on stage and grade. The dotted line indicates the cut-off value of Trx1 level (11.4 ng/ml)

As BC shows different anatomical and pathological characteristics according to its stage, the effect of BC stage on the blood level of Trx1 was examined (Fig. 5). Almost half of the patients were at stage 2 (47.2%), showing a Trx1 level of 21.94 ± 6.06 ng/ml. The second most common stage was 1 (34.9%) with a Trx1 level of 21.64 ± 6.48 ng/ml, while stage 3 (14.2%) followed, with a level of 21.86 ± 9.76 ng/ml. Stages 0 and 4 were small in number (1.9% each), since it was difficult to recruit corresponding patients, and exhibited levels of 22.25 ± 7.47 and 28.97 ± 6.39 ng/ml, respectively. When stage 1 and 2 were combined, they comprised more than 80% of the patients and averaged a blood level of Trx1of 21.79 ± 6.27 ng/ml. All the blood levels of Trx1 from different stages of BC were higher than the cut-off value, indicating that the blood level of Trx1 was not affected by the stage of BC. It was interesting that all stages showed similar levels of Trx1, whereas we hypothesized that the level of Trx1 would increase as stage increases. This interpretation was supported by an R^2^ value of 0.001.

Almost half of the BC patients were in grade 2 (*n* = 51, 48.1%), with a Trx1 level of 21.19 ± 5.87 ng/ml. The levels of Trx1 of grades 1 and 3 were also higher than the cut-off value. More than two third of the patients were in grades 2 and 3, and a large portion of them were likely to have relatively fast-growing and metastatic cancer cells. Altogether, the blood level of Trx1 in BC was not affected by cancer stage or histologic grade.

### Comparison analysis with mammography

**Fig. 6:**
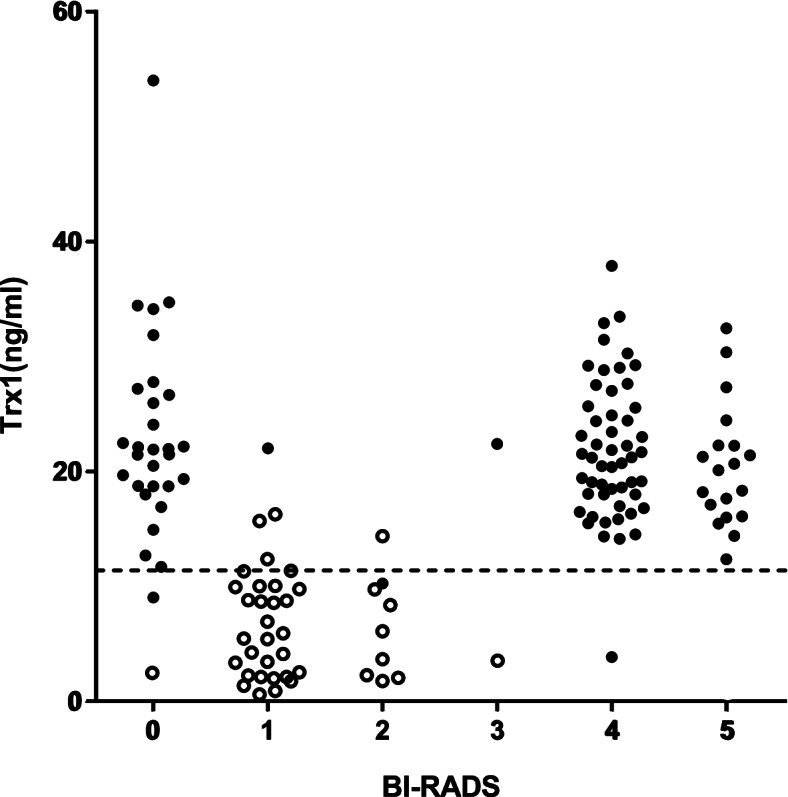
Complementary effect of blood Trx1 level on mammography for higher accuracy. BI-RADS category of subject’s mammogram and corresponding blood Trx1 level were analyzed together. A total of 103 biopsy-confirmed BC patients, and 42 women without cancer who whose mammograms were archived at CNUH were analyzed. The numbers on the X-axis indicate the categories of the BI-RADS scoring system. The dotted line indicates the cut-off value of Trx1 level (11.4 ng/ml). Filled circle (●), biopsy confirmed BC patients; empty circle (○), women without cancer. +

As mammography has long been regarded as the gold standard for the screening of BC, it was necessary to see how well the level of Trx1 corresponded to the matching mammogram. The Trx1 levels and mammograms of 103 out of the 106 participating BC patients and 42 out of the 114 women without cancer were matched and analyzed. As mentioned earlier, BC patients confirmed by biopsy were recruited first, and their mammograms from CNUH were retrieved later. Therefore, retrieval of mammograms was not intentionally attempted to summon participants to get better results. Many of the women without cancer used to undergo mammography at other specialized private clinics during their annual physical examination, and thus their mammograms were not accessible in this study. Therefore, only those whose mammograms were archived at CNUH were selected. This was how testing groups for this comparison study were formed (Fig. 6, and Table 3).

**Table 3:** Comparison study with mammography and Trx1 level

**Variable**	**BI-RADS**	**Trx1 (95% CI)**	**No.**	**%**
Breast cancer patients^a^	0	23.22 (19.89 to 26.55)	29	28.2
(*n* = 103)	1	22.02 (NA)	1	1
	2	10.26 (NA)	1	1
	3	22.38 (NA)	1	1
	4	21.84 (20.14 to 23.53)	52	50.5
	5	20.43 (17.87 to 22.99)	19	18.4
	6	0(NA)	0	0
Women without cancer^b^	0	2.53(NA)	1	2.4
(*n* = 42)	1	6.16(4.49 to 7.82)	32	76.2
	2	6.10(2.34 to 9.87)	8	19
	3	3.61(NA)	1	2.4
**Variable**		**Confirmed BC patients**	**Women without cancer**	
Mammography	BI-RADS 4-5	71	0	
	BI-RADS 0-3	32	42	
	Total	103	42	
Trx1	≥ 11.4 ng/ml	100	5	
	< 11.4 ng/ml	3	37	
	Total	103	42	
Mammography + Trx1^c^	Positive	101	0	
	Negative	2	42	
	Total	103	42	
**Variable**		**Sensitivity(%)**	**Specificity(%)**^**d**^	
Mammography		68.93	100.00	
Trx1		97.09	88.10	
Mammography + Trx1		98.06	100.00	

^a^ BC patients were selected first by their biopsy results, and their matching mammograms were retrieved afterward for analysis. There was 103 out of the 106 participating BC patients

^b^The women without cancer was classified according to BI-RADS score. Among the candidates (*n* = 114), only those whose prior mammograms were archived at CNUH were selected (*n* = 42)

^c^ In combined tests with mammography and Trx1 level, it was regarded as positive when the test results showed the BI-RADS category over 4 or the Trx1 level was higher than the cut-off value (11.4 ng/ml). Similarly, it was regarded as negative when either result was in BI-RADS categories 1-3 or lower Trx1 level than the cut-off value

^d^ It is impossible to get 100% specificity in the real world. However, the test set in the present study showed mathematically perfect results due to the relatively small number of women without cancer, which indicates the potential of the combined tests

The subsequent analysis of mammograms of biopsy-confirmed BC patients was conducted according to the BI-RADS scoring system. Results showed that 71 out of the 103 biopsy-confirmed BC patients were classified into BI-RADS categories 4 (*n* = 52) and 5 (*n* = 19), and the remaining 32 were classified into categories 0 (*n* = 29), 1 (*n* = 1), 2 (*n* = 1), and 3 (*n* = 1). Therefore, 31.1% of biopsy-confirmed BC patients were classified into BI-RADS category 0 to 3, and most of them were in category 0 (*n* = 29, 28.2%). All subjects of women without cancer were classified as BI-RADS category 3 and under, and most of them were in category 1 (*n* = 32, 76.2%). If BI-RADS category 4 and over indicates true positive diagnosis of BC, only 68.9% of the BC patients were initially correctly identified by mammography, in contrast to the high accuracy in identifying women without cancer. When the blood Trx1 level was analyzed, 100 out of the 103 biopsy-confirmed BC patients showed values higher than the cut-off value (11.4 ng/ml), indicating true positive diagnosis of BC. For the women without cancer, 37 out of the 42 showed a Trx1 level below the cut-off value, implying true negative diagnosis of BC. Next, the sensitivity and specificity of mammography and the Trx1 test of biopsy-confirmed patients was estimated by ROC curve analysis. Mammography exhibited a sensitivity and specificity of 68.9 and 100%, respectively. Those of the Trx1 test were 97.09 and 88.10%, respectively. Interestingly, when mammography and the Trx1 test were combined, the sensitivity and specificity rose to an almost perfect 98.06 and 100.0%, respectively. Even though the perfect specificity result should be viewed with caution in light of the relatively small numbers of subjects, it implies that combined examination with mammography and the Trx1 test could create a synergy and achieve very high diagnostic performance.

## Discussion

Currently used tumor biomarkers related to BC diagnosis and management are CA15-3, CA27.29, CEA, and CA125 [21–23]. They can be used to monitor the progression of cancer, evaluate the outcome of a specific treatment, or monitor a recurrence. However, tumor biomarker tests have their own limitations; the level change of the biomarker is not always caused by cancer, and healthy subjects quite often also show increased levels of certain biomarkers. Therefore, tumor biomarker tests cannot be used alone to evaluate or manage BC. Despite the clinical benefits of using tumor biomarker tests to assist in the correct reading or compensate for the technical limits of imaging examinations, it is difficult to find examples with sufficiently high sensitivity and specificity.

In mammalian cells, Trx1 is involved in the regulation of reactive oxygen species (ROS) levels [24, 25]. As it plays a role in the regulation of cellular redox homeostasis, Trx1 has multiple functions in the cell. Therefore, Trx1 is an important entity that is potentially related to the onset of many diseases, including cancer, inflammation diseases, heart failure, and so on. Trx1 has been known to play an important role in regulating cancer cell growth by modulating the DNA binding activity of transcription factors [25–29]. It has been reported that the blood level of Trx1 was specifically higher in breast cancer compared with a few other cancer types [17]. Even though Trx1 is likely expressed in a few different types of cancer as well as in women without cancer, the largest difference in the Trx1 level between sera from women without cancer and cancer subjects was shown in BC. Therefore, if this Trx1 level difference could be distinguished from the difference between levels of women without cancer and other types of cancers, it would be possible to detect BC from the blood. This study focuses on clinical utility in early diagnosis, which is clearly defined by WHO guidelines as being different from screening [16]. Early diagnosis is the recognition of symptomatic cancer in patients in order to facilitate diagnosis and treatment without delay, whereas screening is the identification of asymptomatic disease in an apparently healthy target population. Therefore, in the present study, biopsy-confirmed BC patients were recruited to see how effectively Trx1 level could identify BC as a possible tool to help physicians and patients to proceed through the diagnostic interval of early diagnosis without delay. In this kind of study, other types of cancer were not necessarily examined, for the target group was women with symptomatic BC. Nonetheless, the study of other types of cancer was also carried out to predict how specific the Trx1 test was to BC. In order to expand utility of the Trx1 test to apply to other steps of diagnosis, a study with a much larger size of other cancer types should be carried out.

When the cut-off value was set at 11.4 ng/ml of Trx1, the sensitivity and specificity for distinguishing BC patients from the group of women without cancer plus the group of patients of other cancer types was 97.17 and 94.15%, respectively, with an AUC of 0.990. These values were relatively high compared to previously reported values for other protein cancer biomarker tests, which ranged from 60 to 90% [21–23]. The Trx1 level of patients with other types of cancer was close to that of women without cancer, indicating that the blood Trx1 level has potential to could discern BC from other types of cancer. Even though the sample numbers of certain other types of cancer were insufficient, it still provides important clues about what could be expected from those other types of cancer. It should be acknowledged that there is a pile of reports showing increased expression of Trx1 in various types of cancer [30–35]. However, most of the studies were carried out in cancer cells or tissue, not in blood, and focused on the gene expression level of Trx1. Since the physiological environment of blood is quite different from that of cells or tissue, it is necessary to scrutinize the level of Trx1 protein in blood from large numbers of patients with different types of cancer. The effect of many chronic inflammation diseases was also tested, and they did not have any meaningful effect on the ability of the Trx1 level to identify BC patients (data not shown). Although the total number of subjects with each of the chronic inflammation diseases (e.g., asthma, rheumatoid arthritis, chronic obstructive pulmonary disease, psoriasis, Sjögren’s syndrome, sarcoidosis, diverticulitis, diabetic neuropathy, and immune bowl disease) was low, the average level of Trx1 levels in their blood was lower than the cut-off value.

In this study, the serum was prepared after letting the blood mature for 6 hours, which is not done in the conventional serum separating method. As mentioned earlier, the detection antibody of the ELISA kit used in this study had a very distinct characteristic of a higher affinity to dimeric Trx1 than to monomeric. On the other hand, the counterpart of the detection antibody, the capture antibody, did not show a preference for a specific Trx1 form. As time passed, the form of Trx1 in blood samples shifted from monomers to dimers, possibly through oxidation [36]. Therefore, we have optimized the test system to improve the detection of Trx1: After a series of tests, it was determined that maturing the blood for 6 hours before separating the serum was best to achieve the highest detection of Trx1 from the blood of women without cancer and cancer patients. Spending 6 hours to prepare the test sample seems excessive for a point-of-care environment. However, diagnosis of BC is a series of processes that require both time and diverse tests to reach a final judgment. As long as the forms of Trx1 in the blood of women without cancer and in cancer patients change in a similar pattern, it is still reasonable to test the blood level of Trx1 in this way. In the meantime, an improved kit is in development that will shorten maturation time dramatically. It is interesting to have a pair of antibodies that can differentiate monomeric and dimeric forms of Trx1; it seems possible to develop a kit that quantitates the states of Trx1 in blood samples to understand their role in BC.

Breast cancer patients were identified by the level of Trx1 in sera, and this was not influenced by age. In particular, BC patients in their late 40s to early 50s, the age group with the highest BC incidence in Korea, were differentiated from women without cancer. Breast cancer patients in other age groups were also equally well identified by Trx1 level. It has also been reported that there is no influence of the age and menstrual status of Caucasian women on Trx1 level [17]. Even though the study was conducted with a laboratory-developed ELISA kit with preliminarily produced antibodies, it proved the potential of the Trx1 level to identify BC. Since it has been commonly accepted that the incidence rate of BC gets higher in older age [2, 3], it could be expected that the Trx1 level would follow suit. However, no significant difference was observed.

Breast cancer has complicated classifications and exhibits its own pathological characteristics for diagnosis and treatment as well [37, 38]. IDC, which comprised the largest number of BC patients in the study and in the Korea breast cancer incidence database, had an average Trx1 level of 22.00 ± 7.04 ng/ml. ILC and MC also showed similar values, indicating that blood Trx1 level can be used to detect BC regardless of BC type. The number of cases of DCIS, IMC and ITC were low, since the incidence rate of each was low and, thus, it was difficult to collect blood from these types of BC patients. It will be interesting to conduct a study with a large numbers of DCIS blood samples to check whether the level of Trx1 is higher than the cut-off. If the level of Trx1 can determine BC even in large number of DCIS cases, it will be useful for the early detection of BC. Despite different pathological characteristics of different types of BC, there was no significant difference between Trx1 levels in any type. This strongly indicates that the blood level of Trx1 is a good means to detect BC.

As it was likely that the blood level of Trx1 was suitable to detect BC, it was intriguing to check whether it could differentiate the stage of BC. The level of Trx1 in blood was not significantly changed by the stage of BC. It showed higher values than the cut-off value no matter what the stage of BC was, thus indicated that the Trx1 level was likely to detect BC regardless of BC stage. It was promising to see high accuracy to identify patients from stage 1 as well as other stages. This result means that the blood level of Trx1 could be an alternative modality for the early diagnosis of BC.

The relatively low sensitivity (68.9%) and high specificity (100.0%) of mammography of BC in this study is typical when compared with other recent reports on mammography [11, 13]. It can be argued that the number of women without cancer in this comparison study was too low, resulting in the result of perfect specificity. The reason for including only 42 out of the original 114 subjects of women without cancer in this study was the accessibility of matching mammograms. In Korea, women can visit any specialized private clinic to undergo mammography for their annual physical examination, and thus the mammograms archived in other clinics are not accessible from CNUH. Therefore, the selection of only a small number of the women without cancer participating in this comparison study was not intentionally designed. Because of this situation, some degree of specificity and sensitivity of the Trx1 test was lost, whereas the specificity of mammography was a perfect 100% value.

The fact that more than one-third of biopsy-confirmed BC patients in this study were judged as an inconclusive (BI-RADS category 0) is concerning. The BI-RADS category 0 means that the final assessment of the mammogram is likely to be held off until additional tests and images are available. It may cost money, time, and anxiety, especially if it causes a delay in diagnostic interval of early diagnosis. It is worth noting that the Trx1 test was generally superior to mammography in this study, and that the combined test with mammography and Trx1 showed the highest accuracy to detect BC. Since biomarker tests cannot be used alone to diagnose a certain cancer or disease, and mammography has not achieved desirable performance for BC screening, the combined test will yield complementary effects that mitigate the weakness of each test and provide the highest accuracy for BC detection.

Although there is no doubt of the need to carry on a larger population study with different types of cancer, it is likely that the level of Trx1 can detect BC specifically. In addition, the Trx1 level was not affected by the different characteristics of BC. This indicates that the blood level of Trx1 has potential as a biomarker for BC. Although there have been many reports regarding the role or mode of action of Trx1 in cancer, it has not been completely elucidated [39–43]. When cancer cells grow, the microenvironment of cancer cells is in a hypoxic state that favors ROS generation, resulting in higher oxidative stress. Cancer cells are inclined to protect themselves from oxidative damage via maintaining their redox status to survive and metastasize to distant organs. In this condition, Trx1 modulates redox signaling pathways via thiol-disulfide exchange with redox-responsive proteins, such as the transcription factors Ref-1 and NF-κB, MAP3K5/ASK1, and the Trx1 interacting protein. This kind of signaling causes modulation of cell kinetics, such as activation of proliferation, inhibition of apoptosis, and facilitation of metastasis of cancer cells. However, most previous studies have been done with cancer cell lines or cancer tissue, both of which have an environment very different to the blood system, especially in terms of redox status. It has been reported that the mRNA level of the Trx1 gene is correlated with the proceeding of BC stages, whereas the protein level of Trx1 in blood is likely kept constant [17]. It can be assumed that Trx1 is also likely involved in other cell kinetics, such as the initiation or switch mechanism of BC. A well-designed elaborate study is required to prove this kind of speculation.

It has been long accepted that mammography contributes to improved screening of BC, thus lowering its mortality. Nevertheless, it has some well-known limitations, such as lack of mobility, radiation exposure, uncomfortable personal experience, and cost in certain countries. In addition, satisfactory sensitivity and specificity of mammography, especially for women with dense breasts, has not been achieved. Therefore, it would be greatly beneficial to have a simple, economic, and complementary means to detect BC with high accuracy in the early diagnostic interval. When the Trx1 level of patients was analyzed along with first readings of mammograms from the same patients, the sensitivity and specificity of coupled tests went up to almost perfect levels. Interestingly, this benefit was more obvious in the case of dense breasts (manuscript in preparation). Therefore, it seems that the level of Trx1 in blood could be a promising modality to detect BC as a complement to current diagnostic methods.

## Conclusions

As has long been asked for, the level of Trx1 in serum is likely to be a promising candidate for a better way to detect BC, in concert with mammography, in the early diagnosis process. It can differentiate BC patients from women without cancer and those with other types of cancer with high sensitivity and specificity, regardless of age or pathological and molecular BC types. When it was used together with mammography, it showed almost perfect accuracy to identify BC. Even though it is necessary to study a much larger population of subjects with BC and other types of cancer, it is likely that the level of blood Trx1 can be a novel means to identify BC in the early diagnosis diagnostic interval, as has been requested by WHO.

## Data Availability

The datasets used and/or analyzed during the current study are available from the corresponding author on reasonable request.

## Abbreviations

BC: Breast cancer
Trx1: Thioredoxin 1
ER: Estrogen receptor
PR: Progesterone receptor
HER2: Human epidermal growth factor receptor 2
CA15-3: Cancer antigen 15-3
CEA: Carcinoembryonic antigen
CA27.29: Cancer antigen 27.29
CA125: Cancer antigen 125
ELISA: Enzyme linked immunosorbent assay
IDC: Invasive ductal carcinoma
DCIS: Ductal carcinoma in-situ
ILC: Invasive lobular carcinoma
IMC: Invasive micropapillary carcinoma
MC: Mucinous carcinoma
ITC: Invasive tubular carcinoma
ROC: Receiver operating characteristic
AUC: Area under curve
ROS: Reactive oxygen species
TNBC: Triple negative breast cancer
